# Phospholipase D2 loss results in increased blood pressure via inhibition of the endothelial nitric oxide synthase pathway

**DOI:** 10.1038/s41598-017-09852-4

**Published:** 2017-08-22

**Authors:** Rochelle K. Nelson, Jiang Ya-Ping, John Gadbery, Danya Abedeen, Nicole Sampson, Richard Z. Lin, Michael A. Frohman

**Affiliations:** 1 The Graduate Program in Physiology & Biophysics, Stony Brook University, New York, USA; 2 Department of Physiology & Biophysics, Stony Brook University, New York, USA; 3 Biochemistry and Structural Biology, Stony Brook University, New York, USA; 4 The Undergraduate Program in Biochemistry, Stony Brook University, New York, USA; 5 Department of Chemistry, Stony Brook University, New York, USA; 6 Medical Service, Northport Veterans Affairs Medical Center, Northport, NY USA; 7 Department of Pharmacological Sciences, Stony Brook University, New York, USA

## Abstract

The Phospholipase D (PLD) superfamily is linked to neurological disease, cancer, and fertility, and a recent report correlated a potential loss-of-function *PLD2* polymorphism with hypotension. Surprisingly, *PLD2* ^−/−^ mice exhibit elevated blood pressure accompanied by associated changes in cardiac performance and molecular markers, but do not have findings consistent with the metabolic syndrome. Instead, expression of endothelial nitric oxide synthase (eNOS), which generates the potent vasodilator nitric oxide (NO), is decreased. An eNOS inhibitor phenocopied *PLD2* loss and had no further effect on *PLD2* ^−*/*−^ mice, confirming the functional relationship. Using a human endothelial cell line, PLD2 loss of function was shown to lower intracellular free cholesterol, causing upregulation of HMG Co-A reductase, the rate-limiting enzyme in cholesterol synthesis. HMG Co-A reductase negatively regulates eNOS, and the PLD2-deficiency phenotype of decreased eNOS expression and activity could be rescued by cholesterol supplementation and HMG Co-A reductase inhibition. Together, these findings identify a novel pathway through which the lipid signaling enzyme PLD2 regulates blood pressure, creating implications for on-going therapeutic development of PLD small molecule inhibitors. Finally, we show that the human *PLD2* polymorphism does not trigger eNOS loss, but rather creates another effect, suggesting altered functioning for the allele.

## Introduction

The classic Phospholipase D (PLD) isoforms, PLD1 and PLD2, hydrolyze phosphatidylcholine (PC), the most abundant membrane phospholipid, to generate choline and the second messenger signaling lipid phosphatidic acid (PA)^1^. PLD1 and PLD2 have partially distinct and partially overlapping / redundant roles *in vivo*, with current translational interest^2^ for PLD1 being focused on thrombotic disease^3, 4^, cancer^5^, and the immune system^6–8^, and for PLD2, Alzheimer’s Disease^9^, cancer^10^, and influenza virus infection^11^. A recent study reported a negative correlation of a polymorphism in *PLD2*, R172C, with hypertension^12^. This polymorphism is located in a lipid-binding regulatory phox consensus sequence (PX) domain that is conserved in PLD1 and PLD2. For PLD1, the PX domain binds phosphatidylinositol (3,4,5)-trisphosphate, which facilitates its localization to the plasma membrane and stimulates PLD1’s enzymatic activity^13^. Both the PLD1 and PLD2 PX domains have also been reported to mediate interaction with signaling proteins such as epidermal growth factor receptor (EGFR)^14^, facilitating its endocytosis. Arginine, as a basic amino acid, is frequently involved in the binding to negatively-charged polyphosphoinositides and to protein targets via arginine fingers; thus the substitution of cysteine for R172 could alter PLD2’s cellular function.

PLD activity has been proposed to facilitate multiple pathways that function to increase blood pressure, including formation of very low-density lipoproteins (VLDL)^15, 16^, which, following secretion from the liver, are processed to generate low-density lipoprotein (LDL) particles that can be further modified to form oxidized-LDL (ox-LDL), which oppose vascular relaxation and promote atherogenesis^17, 18^. PLD2 function has also been linked to endocytosis of the angiotensin II type 1 receptor (AT1R)^19^, which promotes increased vascular tone and blood pressure via intracellular signaling, and to the production and secretion of aldosterone^20, 21^, which increases blood pressure by stimulating renal water and salt retention. Taken together, these reports provided multiple rationales for the observation that a polymorphism that could affect PLD2 function correlates with decreased blood pressure^12, 22^.

Undertaking exploration of this topic, we uncovered and report here the unexpected observation that mice lacking PLD2 have *increased* blood pressure, and delineate the pathway through which PLD2 normally functions to lower blood pressure. Ultimately, we propose that the human R172C polymorphism creates a mutant protein with only partial loss of function or altered function, rather than PLD2 functional deficiency. Our findings have implications for therapeutic use of PLD2 inhibitors in other settings; the potential for increased blood pressure will need to be assessed and considered in terms of the risk and potential benefits.

## Results

### PLD2^−/−^ mice have increased systemic blood pressure accompanied by decreased cardiac function

**Figure 1:**
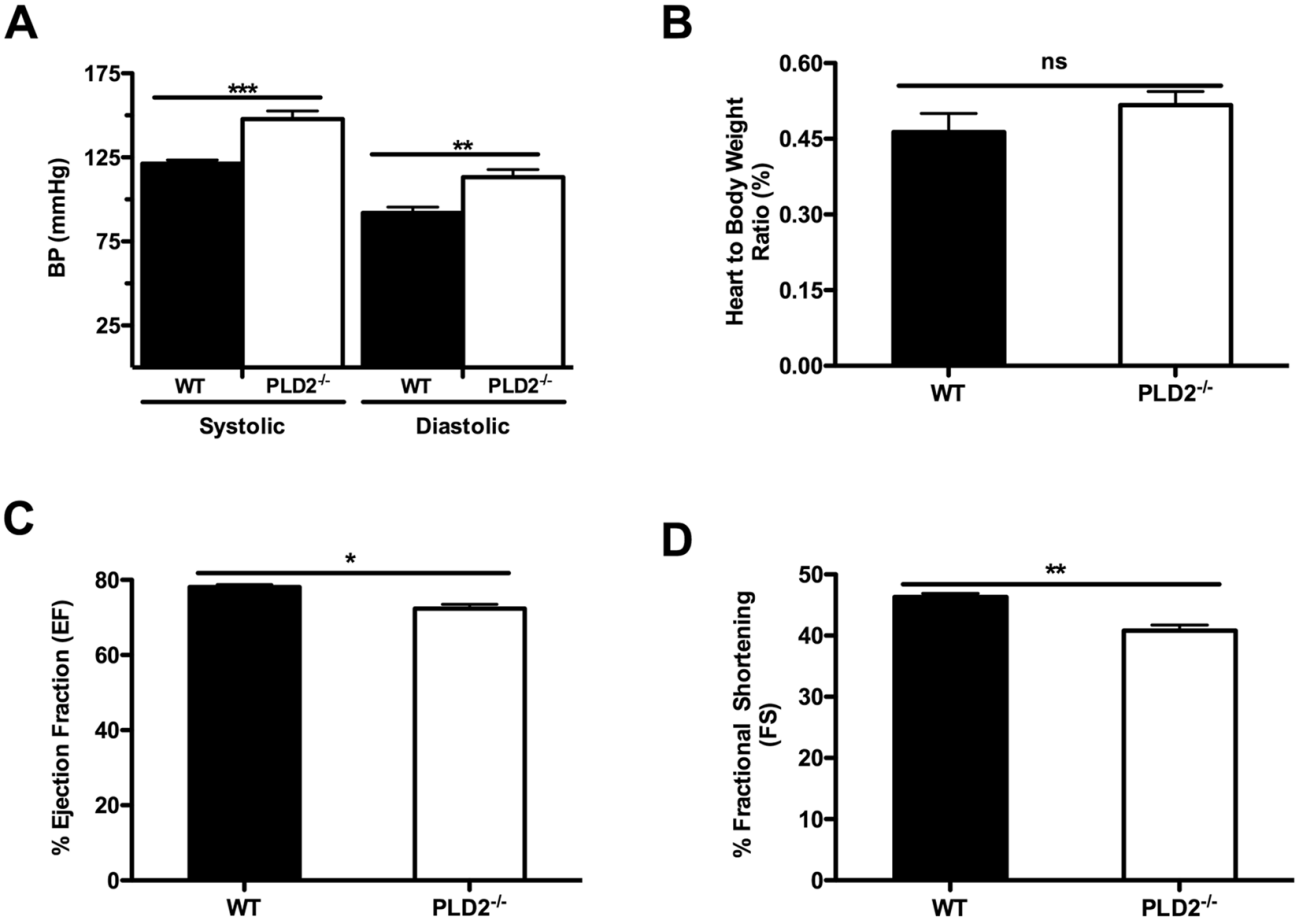
*PLD2* ^−/−^ mice have increased BP and altered cardiac function. (**A**) A non-invasive tail-cuff instrument was used to measure systolic and diastolic blood pressure in 5-month old male mice (n = 6 mice of each genotype; each data point represents the average of 15 measurements) as described in Methods. (**B**) Heart-to-body weight ratio for *PLD2* ^−/−^ and WT mice (n = 3 mice of each genotype). (**C–D**) Echocardiogram sonogram was used to determine fractional shortening (FS) and percent ejection fraction (EF) as described in Methods (n = 3). Mean ± SEM; *p < 0.05; **p < 0.01; ***p ≤ 0.001 (Student’s t-test).

Tail-cuff measurements were used to determine the blood pressure of wild-type (WT) and *PLD2* ^−/−^ mice (Fig. 1A). Unexpectedly, the *PLD2* ^−/−^ mice were found to have increased systolic (∆27 mm Hg; ***P*** = 0.00052) and diastolic (∆21 mm Hg; *P* = 0.0043) BP. This increase is significant in the context of human hypertension, since increases of 20 mm Hg systolic BP/10 mm Hg diastolic BP above the normal range are associated with two- or greater-fold differences in death rates from stroke, ischemic heart disease, and other vascular causes^23^.

One of the major symptoms of hypertension is development of cardiac hypertrophy, since additional ejection force is needed to overcome the increased aortic blood pressure. The heart-to-body weight ratio in *PLD2* ^−/−^ mice was not significantly increased compared to WT mice (Fig. 1B, ***P*** = 0.31). However, cardiac function in *PLD2* ^−/−^ mice as assessed by echocardiogram revealed a significant decrease in the % fractional shortening and ejection fraction (Fig. 1C, D; ***P*** = 0.012, 0.0071), albeit the levels are still within the normal range. Similarly, the left ventricular diastolic volume was decreased by 15% in the *PLD2* ^−/−^ mice (n = 3, ***P*** = 0.025). There were no significant differences in the left ventricular posterior wall thickness, the left ventricular interior diameter, or the interventricular septal thickness; however, the interventricular septal thickness at the end of diastole trended towards being increased (15% increased, ***P*** = 0.074). These decreases, while not indicative of cardiac hypertrophy or failure, are significant and likely reflect a compensatory response (mild adaptive hypertrophy) to the increased blood pressure in the *PLD2* ^−/−^ mice.

### The increased blood pressure in PLD2^−/−^ mice is not associated with obesity or hyperlipidemia

**Figure 2:**
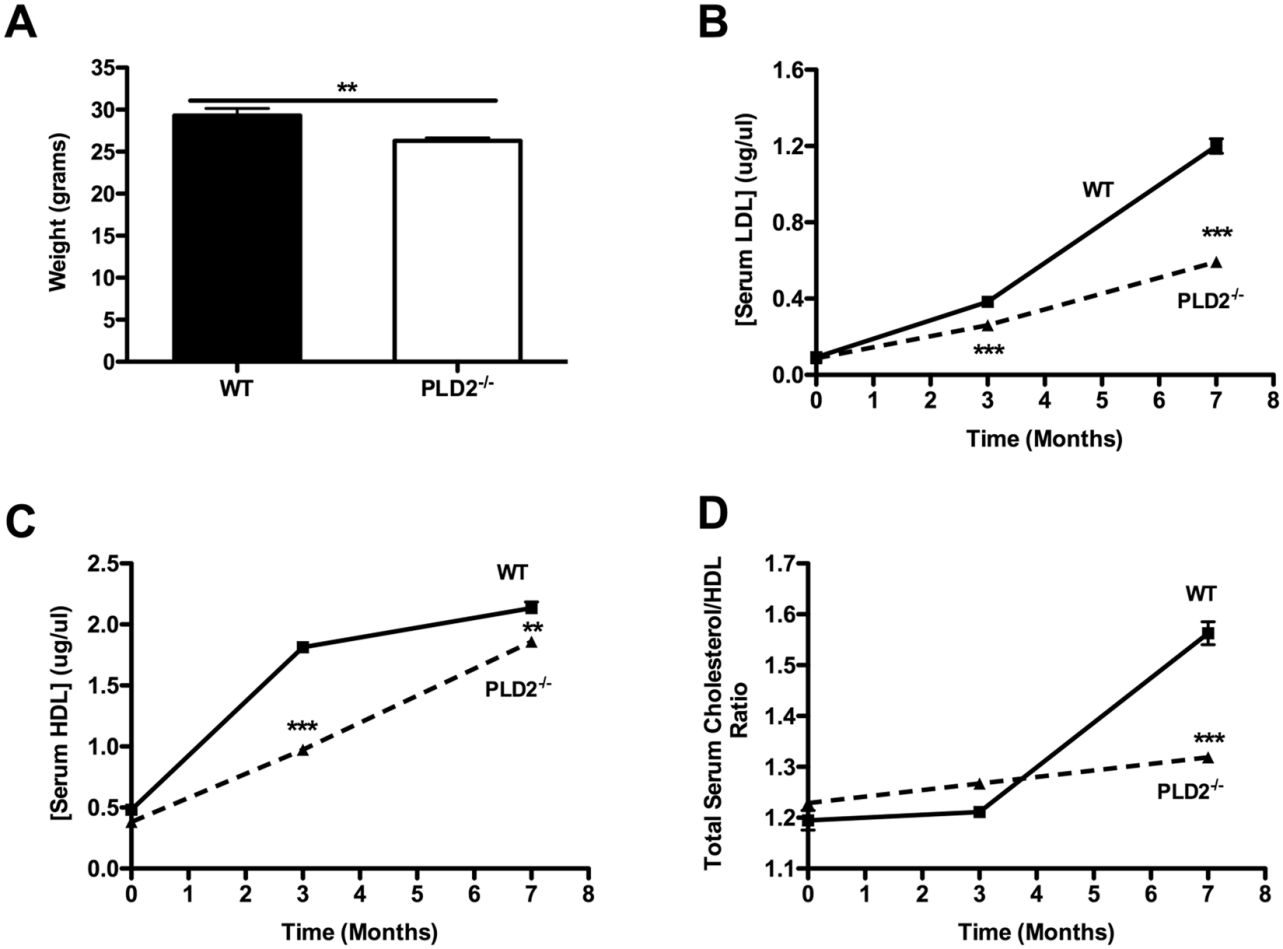
*PLD2* ^−/−^ mice are neither obese nor hyperlipidemic. (**A**) weight at 5 months, n = 7 mice of each genotype. LDL (**B**), HDL (**C**), and total serum cholesterol/HDL ratio (**D**) after 0, 3 and 7 months on a high fat diet (n = 3 mice of each genotype). Mean ± SEM; **p < 0.01; ***p ≤ 0.001 (Student’s t-test).

Hypertension can evolve as a co-morbid disease linked to obesity, HDL cholesterol, and diabetes (the metabolic syndrome)^24^. However, the *PLD2* ^−/−^ mice were found to weigh significantly less than age-matched WT mice (Fig. 2A, ***P*** = 0.0076), and the levels of serum LDL and HDL levels were the same or lower than in WT mice when placed on a high fat diet for 7 months (Fig. 2B [P = 0.00027 at 3 mos and 0.00011 at 7 mos], C [***P*** = 0.000094 at 3 mos and 0.0059 at 7 mos]). The ratio of total serum cholesterol/HDL is used as a risk factor to monitor the development of heart disease^25^; employing this assessment method, *PLD2* ^−/−^ mice would be predicted to be at lower risk of cardiovascular disease than WT mice (Fig. 2D, *P* = 0.00047 at 7 mos). Thus, the increased BP would appear to arise from a mechanism distinct from the ones associated with the metabolic syndrome.

### PLD2^−/−^ aortas have decreased levels of endothelial nitric oxide synthase (eNOS), an effector of vasodilation

**Figure 3:**
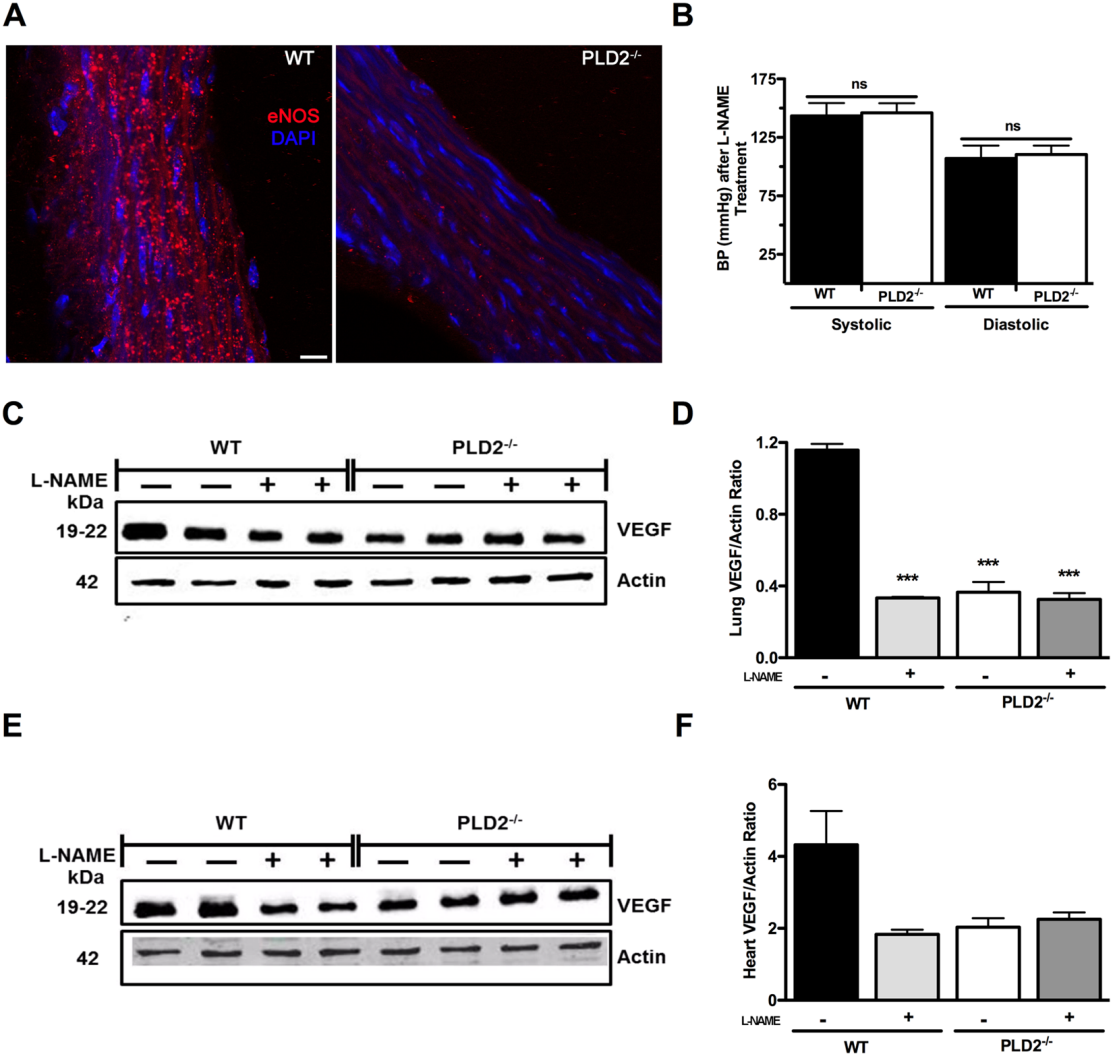
Decreased BP in *PLD2* ^−/−^ mice results from reduced levels of eNOS protein. (**A**) Immunofluorescent staining of eNOS in sections of aortas from WT and *PLD2* ^−/−^ mice. Bar, 7.5 μM. Representative image from 4 mice of each genotype. (**B**) Systolic and diastolic blood pressure following treatment with L-NAME for one week (n = 4). (**C**–**F**) Measurements of VEGF protein levels by western blotting of *PLD2* ^−/−^ and WT mice lungs (**C**,**D**) and heart (**E**,**F**) were normalized to actin expression levels (n = 4 mice of each genotype). A representative blot is shown. The regions of the Western blots containing the VEGF and actin immunoreactive bands were scanned and the relative abundance of the individual samples quantified using an Odyssey CLx imaging system. Mean ± SEM; ***p ≤ 0.001; B: Student’s t-test; D, F: One-way ANOVA with Bonferroni’s Multiple Comparison Test.

In surveying potential causes for the increased BP, we discovered, using immunofluorescent staining, that eNOS protein expression was dramatically reduced in the aorta of *PLD2* ^−/−^ mice (Fig. 3A). Quantitatively, the eNOS fluorescent signal was reduced by 62% (±2.9%, n = 4, ***P*** = 0.00049). Moreover, virtually all of the remaining fluorescence was localized diffusely in the cell rather than in punctate form at the plasma membrane. The activity of eNOS is tightly regulated via several mechanisms including control of its subcellular localization^26, 27^. eNOS is thought to be active primarily when present in specialized plasma membrane domains rich in sphingomyelin, cholesterol, and caveolin, called caveolae^28–30^; cytoplasmic and Golgi-localized eNOS generate relatively little NO and are not activated by signaling events. This finding suggested that aortic endothelial cells in *PLD2* ^−/−^ mice might be failing to generate NO, a potent vasodilator. To test this hypothesis, we treated WT and *PLD2* ^−/−^ mice with the nitric oxide synthase inhibitor N-ω-nitro-L-arginine methyl ester (L-NAME) for a week, which suffices to elicit a significant increase in blood pressure due to eNOS inhibition^31^, and reassessed BP. Systolic and diastolic BP in the WT mice rose by 18% (∆ 20 mm Hg) and 17% (∆ 14 mm Hg), respectively, whereas no increase was seen for the *PLD2* ^−/−^ mice (Fig. 3B vs Fig. 1A). Within one week of L-NAME treatment (Fig. 3B), BP was nearly identical in both the WT and *PLD2* ^−/−^ mice, suggesting that the PLD2 deficiency-induced loss of eNOS and endothelial cell NO production underlies the increased BP in the *PLD2* ^−/−^ mice. Similar outcomes were observed after an additional week of L-NAME treatment. Similarly, L-NAME treatment decreased the % fractional shortening, the % ejection fraction, and the left ventricular diastolic volume in the WT mice but not in the *PLD2* ^−/−^ mice, eliminating the difference between the mouse strains (not significant (n.s.), n = 3). We next examined levels of VEGF in the lung and heart, since VEGF levels have been reported to decrease in some models of hypertension^32, 33^. Consistent with these reports, VEGF levels fell in WT mice after 15 days of L-NAME treatment and the resulting sustained hypertension (Fig. 3C–F; the decrease was highly significant in the lung (***P*** = 0.0002) and almost achieved significance (p = 0.069) in the heart). In contrast, VEGF levels were initially at reduced levels in the *PLD2* ^−/−^ mice and were not affected by L-NAME treatment, suggesting that the PLD2-deficiency induced reduction in VEGF is occurring through an eNOS-dependent mechanism.

### PLD2 knock-down in the human umbilical vein cell line EA.hy926 decreases eNOS expression and NO production

**Figure 4:**
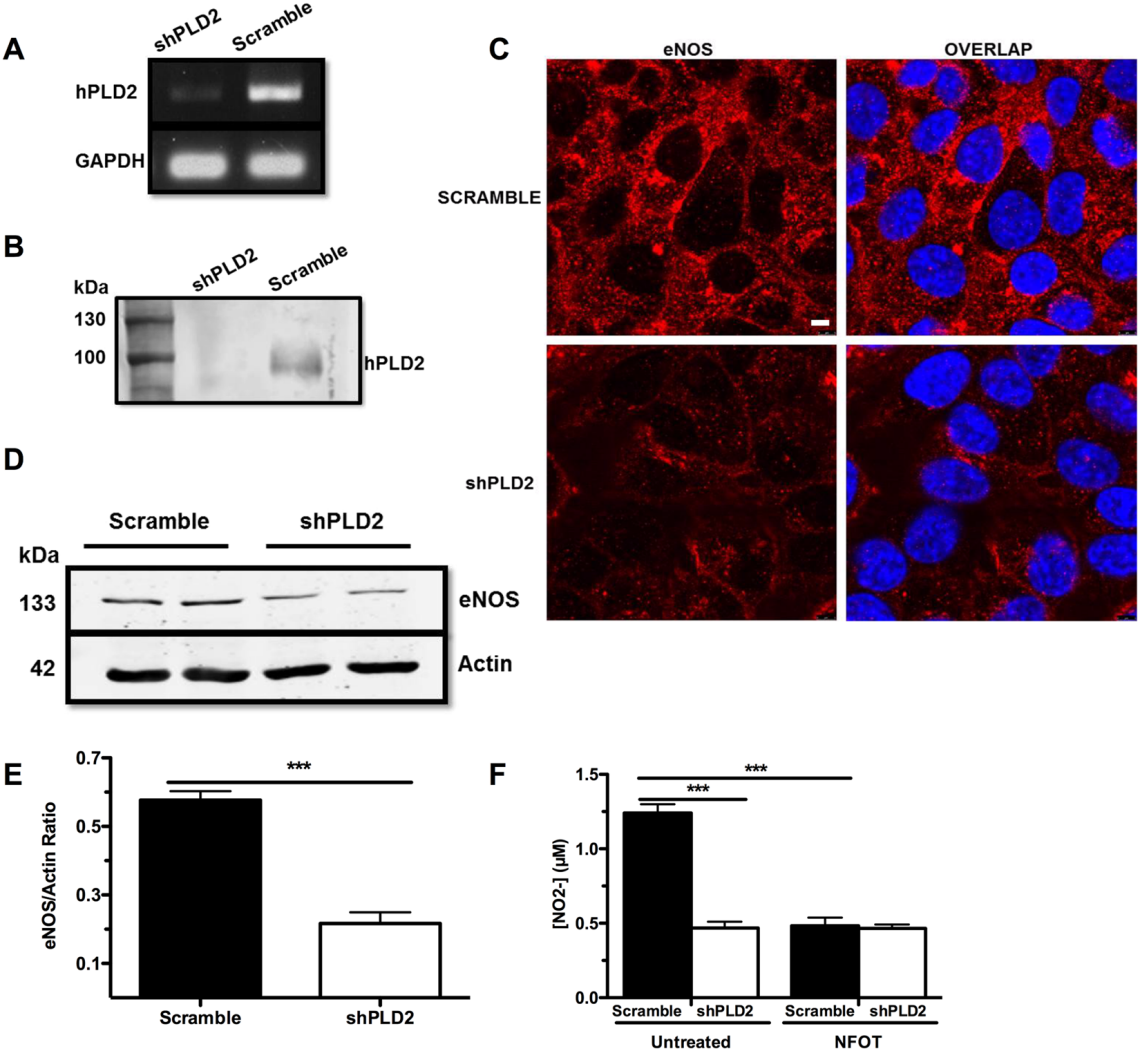
Stable shRNA knockdown of PLD2 in EA.hy926 endothelial cells decreases eNOS protein expression levels and NO production. (**A**,**B**) Stable *PLD2* knockdown cells (shPLD2 cells) were generated using shRNA lentiviral particles. In parallel, control shRNA sequences were used to generate a control cell line (Scramble cells). PLD2 knockdown was confirmed by qRT-PCR (**A**) and western blotting as shown in the cropped gel and blot images, respectively (**B**). Bar, 5 μM. The region of the blot containing the PLD2 immunoreactive band was scanned using an Odyssey CLx imaging system. (**C**) Confocal microscopy of eNOS protein expression as detected by immunofluorescent staining in Scramble and shPLD2 cells. Representative image of 3 experiments. (**D**) Western blotting of eNOS in Scramble and shPLD2 cells, representative blot (n = 3 experiments). The regions of the Western blots (Suppl. Figure 1) containing the eNOS and actin immunoreactive bands were scanned and the relative abundance of the individual samples quantified using an Odyssey CLx imaging system. (**E**) Quantification of western blot with eNOS levels normalized to actin (n = 3 experiments). (**F**) Measurement of nitrate production using a Griess Reaction Kit to quantify eNOS activity with and without treatment with a small molecule PLD2 inhibitor (NFOT) at 10 μM for 24 hours (n = 7 experiments). Mean ± SEM; ***p ≤ 0.001; **E**, Student’s t-test; **F**, one-way ANOVA with Bonferroni’s Multiple Comparison Test.

Since the decrease in eNOS expression in the PLD2^−/−^ mice could have arisen from a long-term compensatory effect or from loss of a scaffolding role due to lack of expression of the protein, we next sought to examine short-term protein loss or inhibition. *In vivo* selective inhibition of PLD2 is not currently practical, since the existing PLD2-selective small molecule compounds have short half-lives^11, 34^ and methods for their continuous delivery have not been established. To address these issues, delineate the mechanism through which PLD2 regulates eNOS expression and to examine eNOS activity directly, we used a commercial lentiviral shRNA approach that employs three different shRNA sequences to establish a human umbilical vein cell line with greatly diminished PLD2 expression (shPLD2). A control lentivirus expressing scrambled shRNA sequences was used to generate the control “Scramble” cell line. The resulting stable shPLD2 pooled cell line exhibited a > 90% knockdown of PLD2 mRNA as assessed by semi-quantitative qRT-PCR (Fig. 4A), and PLD2 protein was undetectable (Fig. 4B). Immunofluorescent confocal microscopy then revealed that PLD2 knockdown substantially decreased eNOS expression, especially on the plasma membrane (Fig. 4C), and quantitative western blotting indicated that the eNOS expression was 62% reduced in the shPLD2 cell pool (***P*** = 0.0010, Fig. 4D,E). Finally, we examined NO production by the Scramble and shPLD2 endothelial cell lines. The shPLD2 cell line generated only 38% as much NO as the Scramble cell line (***P*** = 0.0000021, Fig. 4F). Treatment of the Scramble cells with a small molecule PLD2-selective inhibitor, NFOT^35^, similarly reduced NO production (39% of the control value, as assessed by release of nitrite (NO_2_^−^) into the cell culture media; ***P*** = 0.00000034), but NFOT had no further effect on the shPLD2 cells, confirming that NFOT mediates its effect through inhibition of PLD2 activity. These findings demonstrate that PLD2 regulates eNOS expression in an acute manner, rather than through long-term compensatory changes, and also demonstrate that PLD2 mediates its regulation of eNOS activity through PLD2 enzymatic activity rather than via a scaffolding mechanism, since ablation of the PLD2 protein and simple inhibition of its activity yield the same outcome on NO production. Finally, the effect we demonstrate of the PLD2 inhibitor NFOT to lower eNOS expression levels in endothelial cells suggests that pharmacological inhibition of PLD2 *in vivo* should increase blood pressure and induce compensatory cardiac changes in contractility.

### PLD2 knock-down increases expression of HMG-CoA reductase, a negative regulator of eNOS expression levels

**Figure 5:**
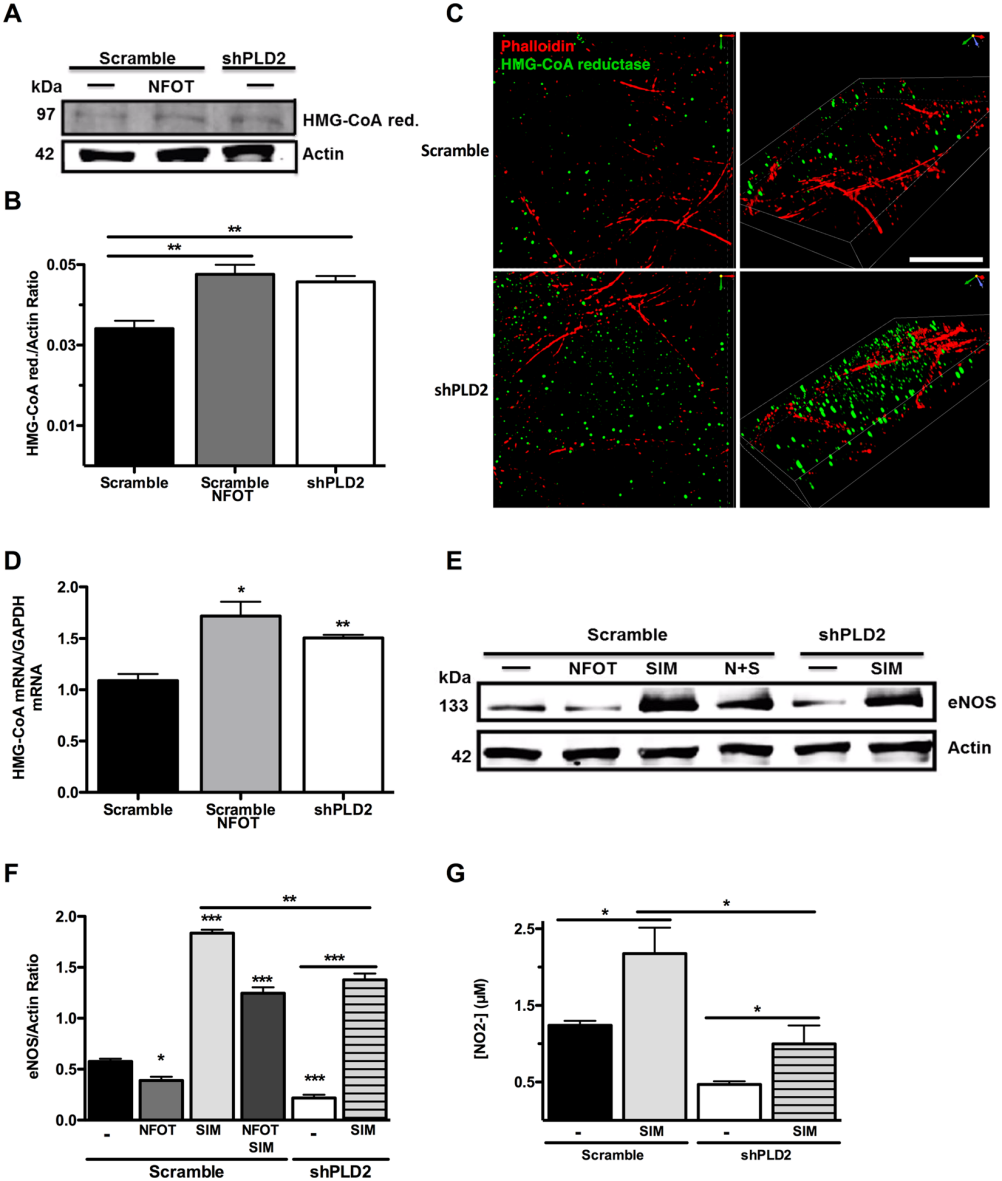
Upregulation of HMG-CoA reductase in shPLD2 cells decreases eNOS expression. (**A**) Representative western blot of HMG-CoA reductase in Scramble and shPLD2 cells. The regions of the Western blots containing the HMG-CoA reductase and actin immunoreactive bands were scanned and the relative abundance of the individual samples quantified using an Odyssey CLx imaging system (**B**) Quantification of western blots with HMG-CoA reductase normalized to actin (n = 4 experiments). (**C**) N-SIM microscopy of HMG-CoA reductase; Bar, 7.5 μM. (**D**) RT-PCR of HMG-CoA reductase in Scramble and shPLD2 cells (n = 3). (**E**) Western blot of eNOS after treatment with NFOT and Simvastatin. Representative image of 3 experiments. The regions of the Western blots (Suppl. Figure 2) containing the eNOS and actin immunoreactive bands were scanned and the relative abundance of the individual samples quantified using an Odyssey CLx imaging system. (**F**) Quantification of western blots with eNOS levels normalized to actin (n = 3). Comparisons without bars made to untreated Scramble values. (**G**) eNOS activity after treatment with Simvastatin (n = 7). Mean ± SEM; *p < 0.05; **p < 0.01; ***p ≤ 0.001; One-way ANOVA with Bonferroni’s Multiple Comparison Test.

HMG-CoA reductase, the rate-limiting enzyme in cholesterol synthesis, has been well studied as a negative regulator of eNOS function, through its production of mevalonate, which decreases eNOS mRNA stability^36^. Statins, which inhibit HMG-CoA reductase activity, have been shown widely to increase eNOS mRNA and protein levels and NO production^37^. Here, using western blotting, we found that HMG-CoA reductase levels were increased by 25% in the shPLD2 endothelial cells (***P*** = 0.0032) and in Scramble cells treated with the PLD2 inhibitor NFOT in comparison to Scramble cells (Fig. 5A,B). N-SIM (structured illumination) microscopy similarly revealed an increase in shPLD2 endothelial cell HMG-CoA reductase expression (Fig. 5C **)**, and qRT-PCR demonstrated increased levels of HMG-CoA reductase mRNA in the NFOT-treated Scramble endothelial cells (***P*** = 0.013) and shPLD2 endothelial cells (***P*** = 0.0048, Fig. 5D).

Next, we tested whether an HMG CoA reductase inhibitor could overcome the PLD2-mediated decrease in eNOS protein expression levels and activity. Scramble endothelial cells treated with the PLD2 inhibitor NFOT had decreased expression levels of eNOS protein (Fig. 5E,F, lanes 1 and 2, ***P*** = 0.012), as described above, whereas treatment with the HMG-CoA reductase inhibitor, Simvastatin, strongly increased eNOS protein expression (lane 3, ***P*** = 0.0000071). The combined treatment was intermediate in outcome (lane 4), with expression being elevated above the Scramble control, but not as high as Simvastatin alone. Similarly, eNOS protein expression was decreased in shPLD2 cells as described above (lane 5, ***P*** = 0.0010), and increased, but not fully, with exposure to Simvastatin (lane 6, ***P*** = 0.0027). Nonetheless, these findings suggest that the PLD2-deficiency-stimulated increase in HMG-CoA reductase expression level and activity underlies the decrease in eNOS protein expression. Finally, we observed a similar outcome through examining NO production: as before, shPLD2 cells generated less NO than Scramble endothelial cells (Fig. 5G, lanes 1 vs 3, ***P*** = 0.000000035), and Simvastatin increased the amounts of NO production in both the control (***P*** = 0.012) and PLD2-knockdown cells (lanes 2 and 4, ***P*** = 0.036), identifying a role for HMG-CoA reductase in the PLD2-deficiency-mediated inhibition.

### Intracellular free cholesterol levels are decreased in PLD2-deficient endothelial cells, and when restored, bypass PLD2 deficiency to elevate eNOS expression

**Figure 6:**
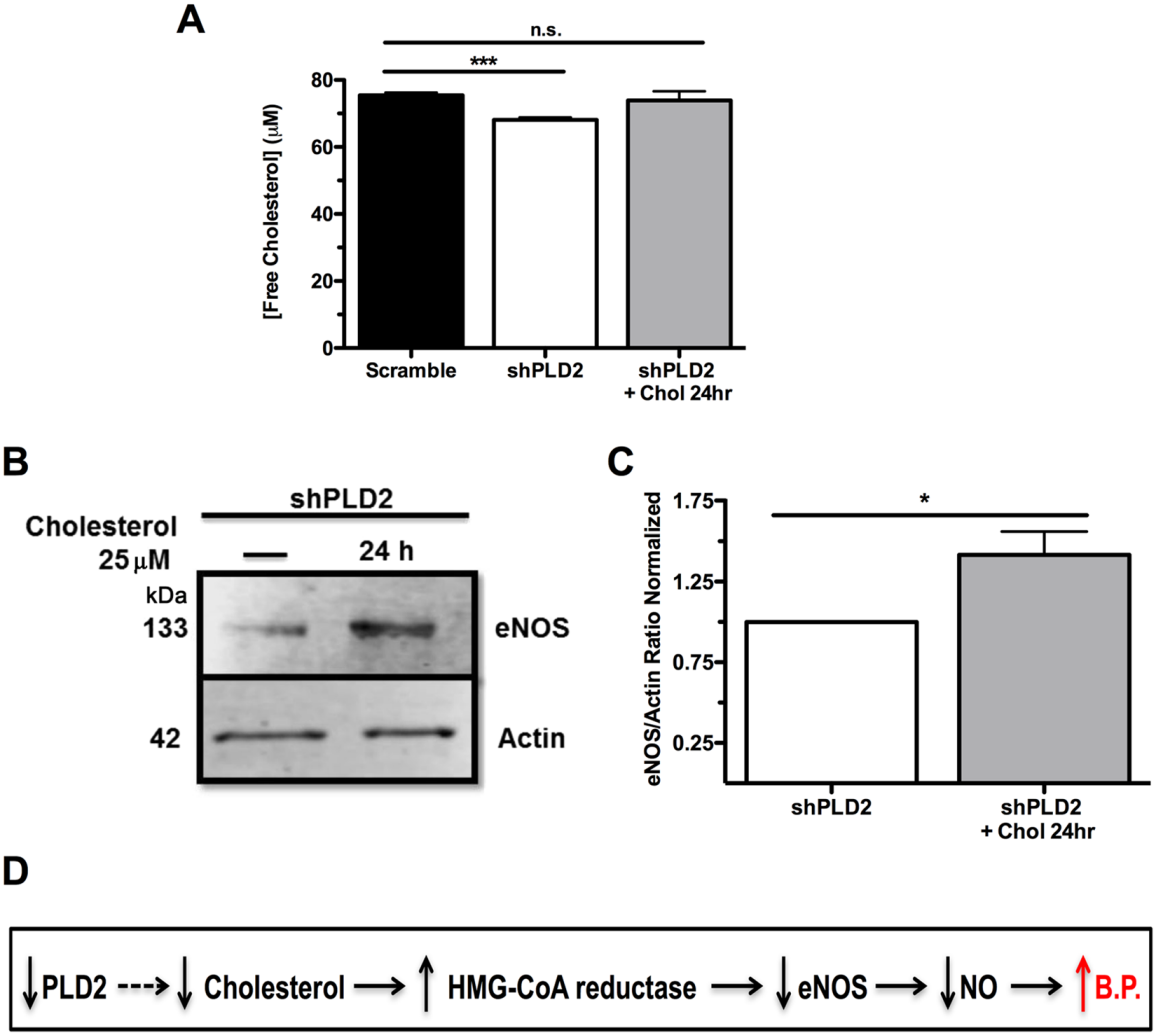
Recovery of eNOS levels in shPLD2 cells following exogenous cholesterol supplementation. (**A**) Free cholesterol levels monitored by cholesterol oxidase assay after culture of cells with 25 μM exogenous cholesterol (in media) for 24 hours (n = 4). (**B**) Representative western blot of eNOS protein in shPLD2 cells with and without cholesterol supplementation. The regions of the Western blots (Suppl. Figure 3) containing the eNOS and actin immunoreactive bands were scanned and the relative abundance of the individual samples quantified using an Odyssey CLx imaging system. (**C**) Quantification of western blot with eNOS levels normalized to actin (n = 5). (**D**) Schematic summary of model. Dotted line indicates that the mechanism involved is unknown at this time. Mean ± SEM; *p < 0.05; **p ≤ 0.001; C, Student’s t-test; A, one-way ANOVA with Bonferroni’s Multiple Comparison Test.

HMG-CoA reductase is regulated both transcriptionally and with respect to activation of the protein by the levels of intracellular free cholesterol^38^. We found that free cholesterol levels are 9% reduced in shPLD2 cells (p < 0.001, Fig. 6A). To establish whether this reduction is biologically significant, we supplemented the shPLD2 cells with 25 μM cholesterol for 24 hours, which increased the intracellular levels by 7%, bringing it almost back to normal levels (***P*** = 0.00092, Fig. 6A). We then examined eNOS protein expression levels, and found that the cholesterol supplementation resulted in a 42% increase (Fig. 6B,C, ***P*** = 0.012). Placing all of these observations together, our findings reveal a novel pathway in which PLD2 deficiency decreases intracellular cholesterol, leading to increased HMG-CoA reductase protein expression and activity, which results in decreased eNOS expression, deceased production of the vasodilator NO, and, finally, hypertension (Fig. 6D).

### R172C confers selective loss of function on PLD2

**Figure 7:**
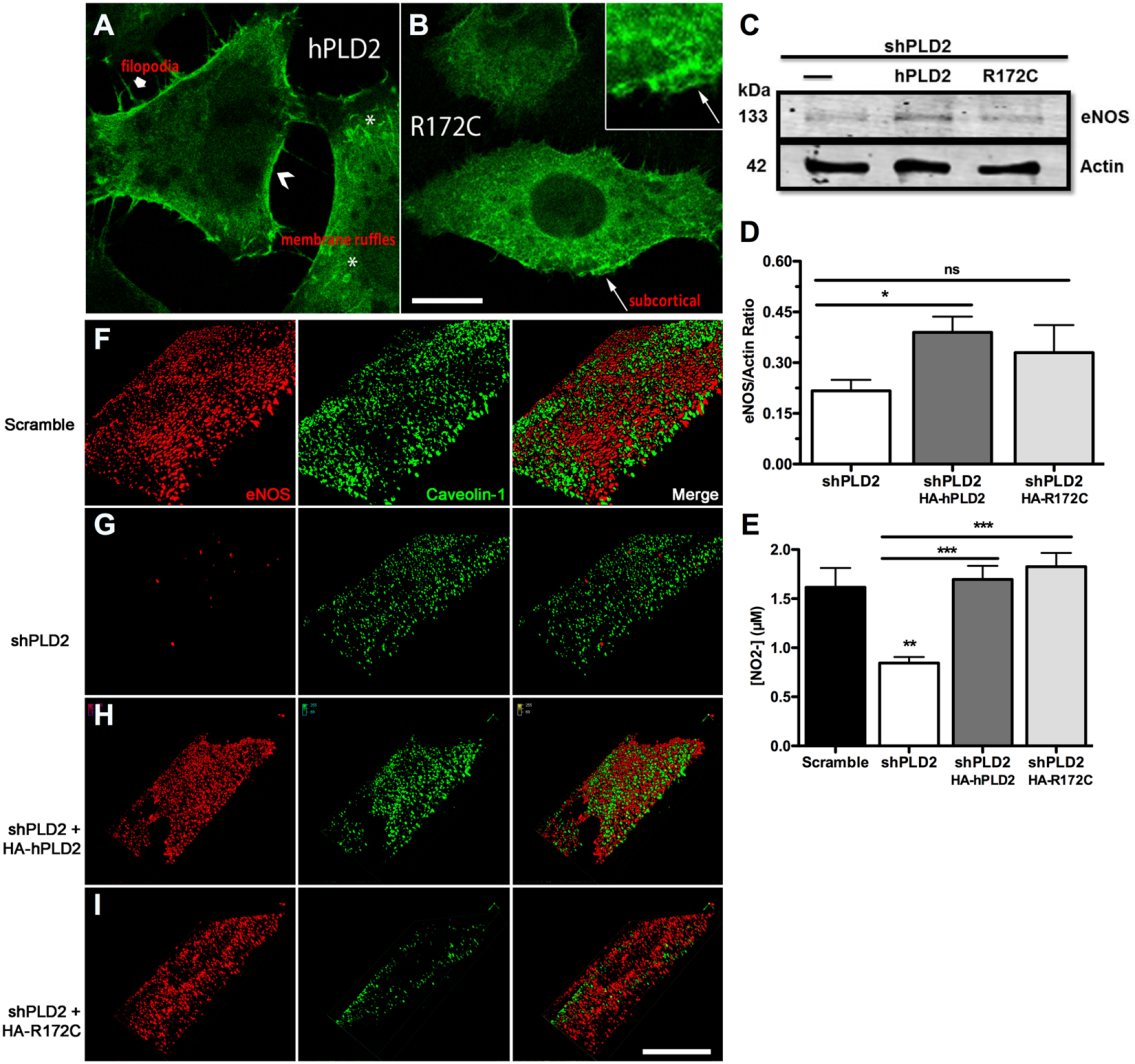
The human PLD2 polymorphism R172C does not alter eNOS signaling but does decrease caveolin-1 protein levels. Overexpression of HA-tagged hPLD2 (**A**) and HA-R172C-PLD2 (**B**) in HeLa cells, visualized using anti-HA immunofluorescent staining (green). Arrowhead, PLD2 localization in filopodia; chevron, in peripheral actin ruffles; *, in dorsal actin ruffles; arrow, in subcortical actin network. Bar, 7.5 μM. Representative image of multiple experiments. (**C**) Representative western blot of eNOS after transfection of HA-hPLD2 or HA-R172C-PLD2 into shPLD2 cells. The regions of the Western blots containing the eNOS and actin immunoreactive bands were scanned and the relative abundance of the individual samples quantified using an Odyssey CLx imaging system. (**D**) Quantification of western blotting with eNOS levels normalized to actin (n = 3). (**E**) eNOS activity as measured by nitrate formation (n = 7). Lane 1 vs 2, ***P*** = 0.0021; lane 2 vs 3, ***P*** = 0.00035; lane 2 vs 4, ***P*** = 0.00012. (**F–I**) N-SIM microscopy of plasma membrane eNOS and caveolin-1. Bar, 7.5 μM. Representative image of multiple Scramble or shPLD2 cells imaged. Cells in H and I were selected for imaging based on expression of HA-hPLD2 or HA-R172C-PLD2 as visualized by anti-HA immunofluorescence in a separate channel (not shown). Mean ± SEM; *p < 0.05; **p < 0.01; ***p ≤ 0.001; One-way ANOVA with Bonferroni’s Multiple Comparison Test.

Returning to the human polymorphism linked to reduced blood pressure^12^, we sought to address whether it functions differently than the wild-type PLD2 allele, since it clearly does not phenocopy PLD2 loss-of-function in mice. Human PLD2 overexpression in cells stimulates F-actin reorganization^39, 40^, as illustrated in Fig. 7A, where both filopodia (arrowhead) and peripheral (chevron) and dorsal (*) membrane ruffles can be observed. In contrast, overexpression of R172C-hPLD2 did not provoke dramatic F-actin reorganization (Fig. 7B). The majority of the R172C-hPLD2 protein appeared to localize to subcortical actin (arrow), in contrast to hPLD2 which localized primarily to the cell cortex. Overexpression of hPLD2 (***P*** = 0.038) and R172C-hPLD2 (n.s., ***P*** = 0.27) increased eNOS protein expression levels in shPLD2 cells (Fig. 7C,D), and more importantly, restored NO production (Fig. 7E). Finally, we examined eNOS and caveolin-1 expression using N-SIM super-resolution microscopy. As before, eNOS protein levels were greatly diminished at the plasma membrane in the shPLD2 cells (Fig. 7F,G), and decreased expression was also observed for caveolin-1, a protein that interacts with PLD2 in lipid rafts in a lipid-dependent manner^41–43^. Overexpression of hPLD2 restored WT levels of expression for both eNOS and caveolin-1 (Fig. 7H). However, overexpression of R172C-hPLD2 rescued only the eNOS expression; no increase in caveolin-1 was observed (Fig. 7I). Together, these findings suggest that the R172C polymorphism does not alter eNOS function; hence, it would not lead to increased BP via this pathway. However, its contrasting effect on caveolin-1 is intriguing, as discussed below.

## Discussion

The regulation of BP is complex, involving numerous physiological and cellular pathways. Supporting roles for PLD2 in this process have been proposed, based on its contribution to the secretion of aldosterone^20, 21^, via facilitation of AT1R signaling^19^, and through promotion of VLDL formation^15, 16^. Based on these studies, the report that a polymorphism in human PLD2 negatively correlates with the development of hypertension appeared to support the hypothesis that PLD2 promotes increased blood pressure and that loss of PLD2 function should result in hypotension. Unexpectedly, we report here that mice lacking PLD2 exhibit hypertension (Fig. 1) as a consequence of reduced levels of eNOS (Fig. 3), which accordingly decreases production of NO (Fig. 4), an important physiological vasodilatory factor.

The proximal cause of PLD2-deficiency-induced eNOS reduction was identified to be a decrease in intracellular free cholesterol (Fig. 6), which stimulates upregulation and activation of HMG CoA-reductase, a well-known negative regulator of eNOS (Fig. 5). The relationship of PLD2 function to intracellular cholesterol levels is poorly understood, but an intriguing possibility arises from a report that PLD2 may be involved in a pathway that destabilizes ABCA1^44^, which mediates the transport of cholesterol and phospholipids from cells to HDL apolipoproteins. PLD2 loss, in this model, would lead to increased ABCA1 protein levels and activity, and consequently, reduced intracellular free cholesterol. This possibility represents an area for future exploration.

Reductions in intracellular cholesterol stimulate both transcriptional upregulation and activation of HMG-CoA reductase, which negatively regulates eNOS mRNA stability and hence protein expression. Simvastatin, an HMG-CoA reductase inhibitor, partially but not fully rescued the decreased eNOS phenotype induced by PLD2 deficiency (Fig. 5), suggesting that HMG-CoA reductase upregulation is only one of the mechanisms affecting eNOS levels in this setting. It is well established that reductions in intracellular cholesterol also suppress eNOS activity^27, 30, 45, 46^ through decreasing the association of eNOS with the plasma membrane in caveolae, where it can be activated by signaling events. Thus, while inhibition of HMG-CoA reductase blocks destabilization of eNOS mRNA, it does not address the underlying cholesterol deficiency, which also impacts eNOS activity.

Conversely, while statins lower blood pressure, and would presumably lower blood pressure in *PLD2* ^−/−^ mice at least in part through increasing eNOS protein expression levels^37^, statins actually mediate numerous effects, including inhibiting the production of specific prenylated proteins, and this may partially underlie the improvement in endothelial function, modulation of immune function, and other pleiotropic cardiovascular benefits associated with statin use. Hence, observing a reduction in blood pressure in statin-treated *PLD2* ^−/−^ mice would not be conclusive evidence for the specific pathway we describe here.

The marked decrease in caveolin-1 in shPLD2 endothelial cells transfected with R172C-hPLD2 is intriguing in that caveolin-1 is a well-known negative regulator of eNOS activity^47^. Reduced caveolin-1 could lead to increased eNOS activity, increased NO production, and vasodilation, consistent with the resistance to hypertension reported to be associated with the human allele^12^.

*In vivo* roles and potential opportunities for therapeutic interventions for PLD2 have been explored in recent years using PLD2^−/−^ mice. PLD2 ablation has been shown to improve outcomes in a model of Alzheimer’s disease^9^, to suppress cancer growth and metastasis in a heterotopic implant model^10^, and to improve outcomes in thrombotic disease models in combination with PLD1 ablation^4^. Finally, PLD2 inhibition has been reported to suppress influenza virus infection of a lung cell line *in vitro* and mouse lungs in an *in vivo* model^11^. While concerns regarding increased blood pressure are of lesser concern in the context of transient inhibition, such as would be performed for management of viral disease and acute thrombotic events, this issue may affect enthusiasm for pursuing PLD2 inhibition in the setting of long-term cancer or neurodegenerative treatment. In particular for cancer, some therapeutics, for example, VEGFR tyrosine kinase inhibitors, are independently associated with a substantially elevated risk of hypertension development^48^, and thus use of a PLD2 inhibitor in parallel with them could result in synergistic adverse effects. Nonetheless, murine studies do not always predict the outcomes of complex signaling pathways in humans. Given the potential utility of PLD2 inhibitors for cancer and acute thrombotic disease, in particular in combination with PLD1 inhibitors^2^, an effect on blood pressure represents a topic that should be assessed carefully if and when PLD2 inhibitors enter the clinic.

## Materials and Methods

### Materials

Simvastatin (Cayman Chemical) was used in culture for 24 hrs at 10 μM, N-[2-[1-(3-Fluorophenyl)-4-oxo-1,3,8-triazaspiro[4.5]dec-8-yl]ethyl]-2-naphthalenecarboxamide (NFOT) (Tocris Bioscience) at 10 μM, and cholesterol (Sigma-Aldrich C8667) at 25 μM.

### Animals

*PLD2* ^−/−^ mice (C57BL/6)^9^, generously provided by Dr. Gilbert di Paolo (Columbia University), were bred in the Stony Brook University animal facilities. Mice were fed standard chow or high-fat diet chow and water *ad libitum* and kept on a 12:12-h dark-light cycle. All experimental procedures were performed in accordance with relevant guidelines and regulations and were reviewed and approved by the Stony Brook University Institutional Animal Care and Use Committee (IACUC). Unless otherwise stated, male mice at 5 mos. of age were studied. Animals were injected with L-NAME (80210; Cayman Chemical) at a dose of 400 µg/g IP once a day for 15 days^31^.

### Non-Invasive Blood Pressure Measurements

Tail-cuff blood pressure (BP) readings were taken at days 0, 7 and 15 of L-NAME treatment using the CODA Multi-Channel, Computerized, and Non-invasive Blood Pressure System for Mice (Kent Scientific). BP measurements were taken twice during the two days leading to each acquisition date to acclimate the mice to the procedure. Blood pressure measurements were obtained by performing 5 acclimation cycles (a cycle = a BP reading) followed by 3 sets of 5 cycles per set.

### Echocardiography

Echocardiogram measurements were taken after 15 days of L-NAME treatment using a Vevo 770 ultrasound device (VisualSonics) with a 30-MHz transducer. Mice were anesthetized with 1% isoflurane and transthoracic echocardiography performed as previously described^49^.

### Cell line creation and qRT-PCR

Human umbilical vein cell line EA.hy926 (CRL-2922; ATCC) was cultured in Dulbecco’s modified Eagle’s medium supplemented with L-glutamine (2 mM), 10% fetal calf serum (FCS), 100 U/mL penicillin and 100 µg/mL streptomycin. The cells were transduced with either Control shRNA Lentiviral Particles-A or with PC-PLD2 shRNA (h) Lentiviral Particles (Santa Cruz Biotechnology), which contain a puromycin-resistance gene to enable selection of stable cell lines or pools. The culture medium was changed 24 hrs following transduction and the cells cultured another day. Puromycin (0.5 μg/ml) was then added for positive selection. The resulting stably-transduced EA.hy926 cell pools were denoted “scramble” and shPLD2, respectively.

### qRT-PCR

Total RNA was extracted with Trizol (Invitrogen). 200–300 ng of total cellular RNA was used with the qScript One-Step SYBR Green qRT-PCR Kit (Quanta Biosciences). Primers used for human HMG-CoA Reductase were: TGTGTGTGGGACCGTAATGG and ACCAAGTGGCTGTCTCAGTG; and for human GAPDH: ACAGTCAGCCGCATCTTCTT and GCATCGCCCCACTTGATTTT.

### Western Blots

*In vivo* samples: Tissues were homogenized in lysis buffer (150 mM NaCl, 1% Triton X-100, 100 mM Tris-HCl, pH7.4, 1 mM EDTA, 1 mM phenylmethylsulfonyl fluoride, 10 μg/ml aprotinin, and 10 μg/ml pepstatin A), ultrasonicated at 4^0^C, microfuged, and supernatants collected. Equal amounts of lysates (20 μg/lane) were separated on SDS-PAGE, transferred to filter paper, and immunostained using mouse anti-VEGF at 1:500 (ab3109; abcam) and rabbit anti-actin at 1:100 (A2066; Sigma-Aldrich). Cell line samples: Cells were cultured in six-well plates until 80–90% confluent, trypsinized, and equal amounts of total protein resolved by SDS-PAGE and blotted onto nitrocellulose membranes (162–0115; Bio-Rad) using NuPAGE Transfer Buffer (NP0006–1; Life Technologies). Membranes were blocked for 1-h in 5% BSA at room temperature (RT) and probed overnight (O/N) with rabbit anti-eNOS at 1:500 (NB300–500; Novus Biologicals), mouse anti-caveolin-1 at 1:500 (sc-53564; Santa Cruz Biotechnology), goat anti-HMG CoA reductase at 1:100 (GTX88456; Genetex), rabbit anti-PLD2 at 1:100 (sc-25513; Santa Cruz Biotechnology), and rabbit anti-Actin at 1:100 (A2066; Sigma-Aldrich) or rabbit anti-GAPDH at 1:500 (sc-25778; Santa Cruz Biotechnology). Membranes were probed with anti-rabbit or anti-mouse secondary Ab at 1:3,000 and developed and quantified using a LI-COR Odyssey Infrared Imager.

With the exception of panel B in Fig. 4, which is shown for illustrative purposes, all of the western blot images shown are provided as representatives of the multiple experiments conducted. The LI-COR Odyssey Infrared Imager quantifies each band based on pixel intensity and generates a pseudo-autorad from the data set. The quantitation presented for all of the western blots (save Fig. 4B) was generated using the raw data returned by the Odyssey imager, not on a scan or digitalization of the autorad shown for illustrative purposes.

### Immunofluorescent Staining and Microscopy

Cryosections (8 μm) of fixed aortas from age-matched male mice on a normal chow diet were stained with rabbit anti-eNOS at 1:200 O/N and for 1 hr with DAPI and Alexa 647 anti-rabbit secondary antibody. Images were captured using a Leica TCS5 Confocal Microscope. Cells were cultured in 12-well plates on glass coverslips until 80–90% confluent, washed with PBS, fixed with 4% paraformaldehyde, permeabilized with Triton X-100, blocked with 5% goat serum for 1 hr, incubated for 1 hr at RT with primary antibodies followed by secondary antibodies and DAPI for another hour, and imaged as above.

### Free-cholesterol measurements

*In vivo* samples: 4–6-week old male mice were placed on a high fat diet chow (HFD) for a total of 7 months. At time points 0, 3 and 7 months, blood was collected retro-orbitally. Serum lipoprotein levels were assessed using the HDL & LDL/VLDL Cholesterol Quantification Kit (K613-100; BioVision) following the manufacturer’s protocol. For cell lines, Scramble and shPLD2 cells were plated in 100-mm tissue culture treated plates until 80–90% confluent, rinsed with PBS, trypsinized and stored at −80 °C until ready for cholesterol extraction. Cholesterol was extracted by lysing the cell pellets in 200 μL 7:11:0.1 chloroform:isopropanol:Nonidet P-40 and incubating at 25 °C for 10 min. The cell debris was removed by centrifugation for 10 min at 15,000 × g and the supernatant collected, dried with a rotary evaporator followed by 30 min in vacuum, and resuspended in 50 μL isopropanol. Cholesterol concentrations were measured using the enzyme cholesterol oxidase^50^, which converts cholesterol to cholest-4-en-3-one, the formation of which can be monitored at 240 nm. Cholesterol samples were added to assay buffer (50 mM sodium phosphate pH 7.0, 0.025% Triton X-100, 0.020% bovine serum albumin) pre-incubated for 10 min with 125 nM cholesterol oxidase at 37 °C. The total reaction was incubated for 45 min at 37 °C. The samples were loaded into a 96-well UV transparent quartz microplate and the absorbance at 240 nm measured using a BioTek Synergy 2 plate reader. A standard curve of known cholesterol concentrations was prepared and measured in parallel to convert absorbance values of each sample to cholesterol concentrations.

### NO Release Analysis into Cell Media

EA.hy926 cells were cultured in 24-well plates in normal growth medium until 80–90% confluent. NO level was assessed in 20 µl of reduced serum medium using a Nitrate/Nitrite Fluorometric Assay Kit (Cayman Chemical) following the manufacturer’s protocol.

### Statistical analysis

Numerical data are presented as mean ± SEM. Student’s t-test was used to compare the differences between two groups and one-way ANOVA with Bonferroni’s Multiple Comparison Test to compare the differences between three or more groups. Significance was based on a value of p < 0.05. Experiments were performed in duplicate unless otherwise noted in the Figure Legends. Statistical analysis was performed on the cumulative average values for each independent experiment (n = repeats [separate experiments]).

### Data availability statement

All data generated or analyzed during this study are included in this published article. No datasets were generated or analyzed during the current study.

## Electronic supplementary material

Supplementary figures
