# Supplementary material for: Phospholipase D2 loss results in increased blood pressure via inhibition of the endothelial nitric oxide synthase pathway
Source: Sci Rep. 2017 Aug 22;7:9112. doi: 10.1038/s41598-017-09852-4 (PMC5567230; doi:10.1038/s41598-017-09852-4)
Supplement: Supplementary file 1 — Supplementary figures [file 41598_2017_9852_MOESM1_ESM.pdf]

**Phospholipase D2 loss results in increased blood pressure via inhibition of the endothelial nitric oxide synthase pathway**

Rochelle K. Nelson, Jiang Ya-Ping, John Gadbery, Danya Abedeen, Nicole Sampson, Richard Z. Lin, and Michael A. Frohman

## Supplemental Data

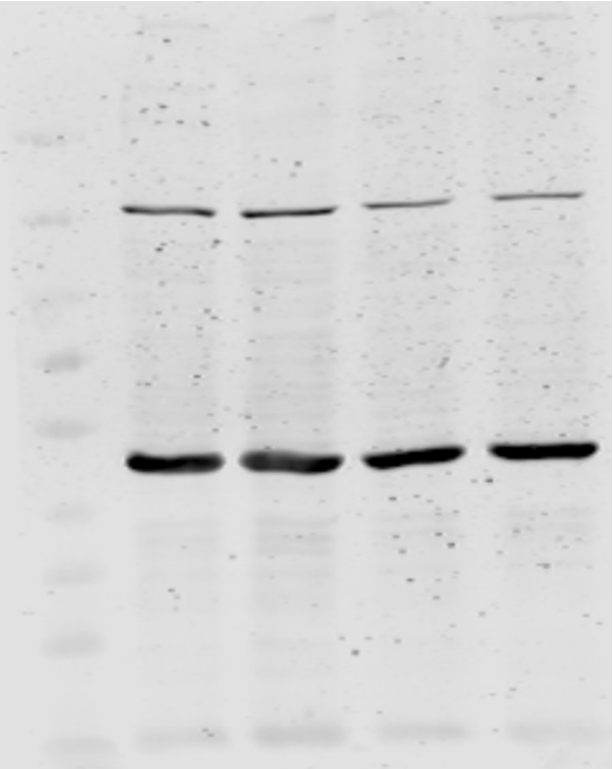

**Supplemental Fig. 1**. Full, original scan of representative western blot image in Fig. 4D. The scan was performed on an Odyssey CLx imaging system, using secondary antibodies labeled with two different infrared dyes to independently visualize and quantitate actin and eNOS. The scan was saved as a black and white image. Supplemental Fig. 2 shows an example of such scans in color. Left lane, markers not shown in Fig. 4D.

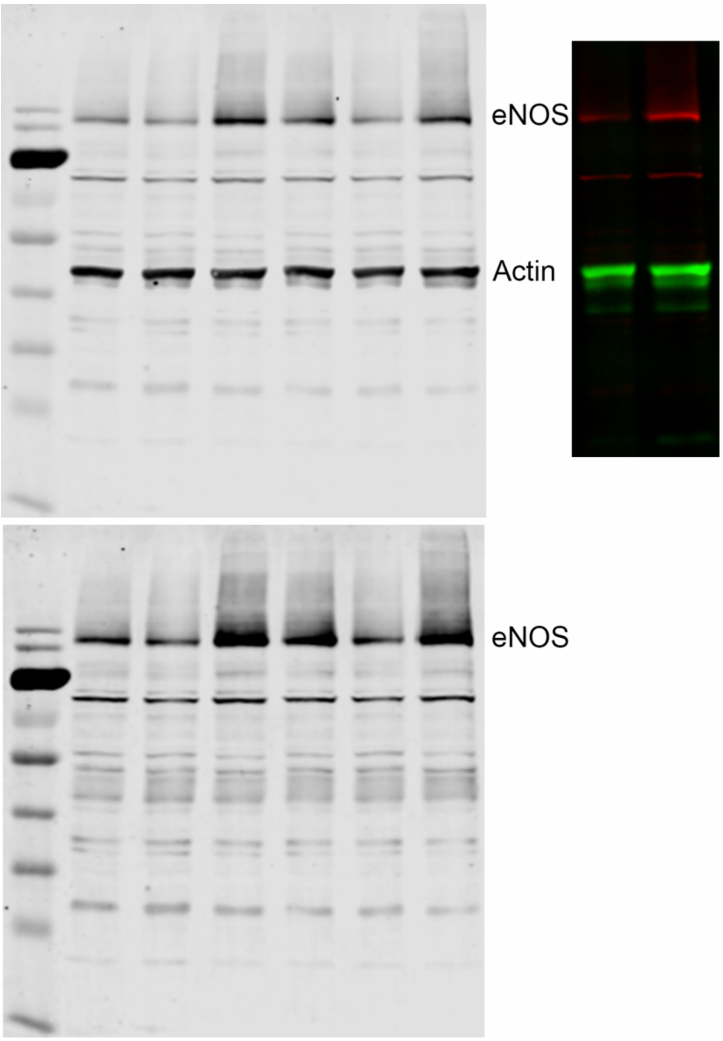


**Supplemental Fig. 2**. Full, original scan of representative western blot image in Fig. 5E. The scan was performed on an Odyssey CLx imaging system, using secondary antibodies labeled with two different infrared dyes to independently visualize and quantitate actin and eNOS. The scan is shown at two different levels of intensity in black and white (the lower scan was used to generate Fig. 5E). The colored image on the right shows the actual two-color image (the two rightmost lanes) as example. Left lane, markers not shown in Fig. 5E.

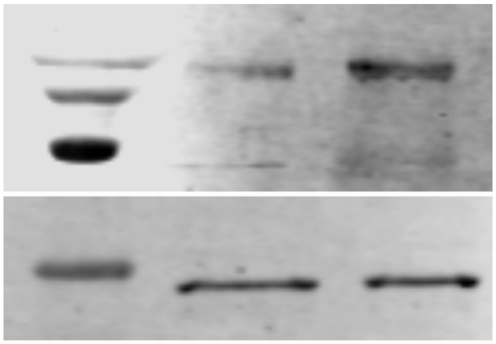

**Supplemental Fig. 3**. Full, original scan of representative western blot image in Fig. 6B. The scan was performed on an Odyssey CLx imaging system, using secondary antibodies labeled with two different infrared dyes to independently visualize and quantitate actin and eNOS. Only a portion of the blot was scanned, from which a cropped section was used to generate Fig. 6B. Left lane, markers not shown in Fig. 6B.
